# Salvage Radiation Therapy for Localised Prostate Cancer Recurrence Following High‐Intensity Focused Ultrasound (HIFU) Failure

**DOI:** 10.1111/1754-9485.70009

**Published:** 2025-08-15

**Authors:** Atsuto Katano, Masanari Minamitani, Subaru Sawayanagi, Hideomi Yamashita

**Affiliations:** ^1^ Department of Radiology The University of Tokyo Hospital Bunkyo‐ku Tokyo Japan

**Keywords:** prostatic neoplasms, radiation oncology, retrospective studies, salvage therapy

## Abstract

**Introduction:**

Prostate cancer is a prevalent malignancy with rising incidence rates globally. High‐intensity focused ultrasound (HIFU) is a promising, minimally invasive treatment for localised prostate cancer. However, recurrence following HIFU, indicated by rising PSA levels or imaging‐confirmed local recurrence, remains a concern. Salvage radiotherapy is a potential treatment for recurrent prostate cancer post‐HIFU, but evidence on its efficacy and safety is limited.

**Method:**

This retrospective study evaluated eight patients with localised prostate cancer who underwent salvage radiotherapy following HIFU failure. Salvage treatment consisted of external beam radiation therapy (EBRT), delivered either as conventional fractionation (76 Gy in 38 daily fractions) or stereotactic ablative radiotherapy (36.25–40 Gy in five alternate‐day fractions). Outcomes assessed included prostate‐specific antigen (PSA) response, biochemical progression‐free survival (bPFS), overall survival (OS) and treatment‐related toxicity profiles.

**Results:**

All eight patients experienced a significant decline in PSA levels, with no biochemical failures observed during a median follow‐up of 72.4 months. The 5‐year biochemical failure‐free survival and overall survival rates were 100%. Among the eight patients, 6 (75%) had Grade 2 or lower genitourinary toxicity, and 5 (62.5%) had Grade 2 or lower gastrointestinal toxicity, with no Grade 3 or higher adverse events.

**Discussion:**

Salvage EBRT after HIFU failure provides durable disease control with manageable side effects. Further research with larger cohorts is needed to refine treatment strategies for recurrent prostate cancer.

## 1 Introduction

Prostate cancer is a common malignancy affecting men globally, with its incidence steadily rising. The number of new cases is projected to increase from 1.4 million annually in 2020 to 2.9 million by 2040, driven by demographic shifts and increased life expectancy [1]. For patients with localised prostate cancer, representative treatment options include active surveillance, radical prostatectomy and radiotherapy [2].

Focal therapy presents a promising, minimally invasive option for the targeted treatment of localised prostate cancer [3, 4]. High‐Intensity Focused Ultrasound (HIFU) is one of the most advanced focal therapy modalities, showing significant progress in research and promising outcomes [5]. HIFU is a non‐invasive technique using a piezoelectric transducer to deliver precise, high‐energy pulses while sparing surrounding tissue [6].

Despite its low rate of adverse events, prostate cancer recurrence following HIFU, indicated by rising prostate‐specific antigen (PSA) levels or imaging‐confirmed local recurrence, remains a concern. Bakavicius et al. [7] conducted a comprehensive literature review of focal HIFU therapy in the MEDLINE database, revealing that clinically significant in‐field recurrence and out‐of‐field progression occurred in 22% and 29% of prostate cancer patients, respectively. In such cases, salvage external beam radiation therapy (EBRT) is recommended to control the disease, according to guidelines issued by the American Urological Association, the American Society for Radiation Oncology and the Society of Urologic Oncology [8].

Currently, there is no standard treatment for patients who experience biochemical failure after HIFU. Salvage radiotherapy is a promising option, but evidence is still limited. This study presented a series of such cases, focusing on treatment outcomes, side effects and overall survival.

## 2 Method

This retrospective study analysed patients at our institution who received salvage EBRT after HIFU for localised prostate cancer, including those treated between September 2009 and December 2020, focusing on treatment outcomes, side effects and long‐term prognosis. Eligible patients met the following criteria: (i) Initially underwent focal HIFU as their primary treatment. (ii) Later developed biochemical recurrence, defined by the Phoenix criteria as a PSA increase of 2 ng/mL above the nadir [9]. (iii) Recurrence was confirmed by biopsy. (iv) No evidence of distant metastases. (v) Sufficient clinical data were available for analysis. All procedures performed in this study were in accordance with the ethical standards of the institutional and national research committee and with the 1964 Helsinki Declaration and its later amendments. The study protocol was approved by the Institutional Review Board (IRB) with approval number [3372‐7].

Salvage EBRT was administered using intensity‐modulated radiotherapy (IMRT) and image‐guided radiotherapy (IGRT), with either a conventional fractionated dose of 76 Gy delivered in 38 daily fractions or a stereotactic ablative radiotherapy (SABR) of 36.25–40 Gy delivered in 5 fractions every other day. The clinical target volume (CTV) was targeted with no prophylactic lymph node irradiation. The CTV determination and the decision regarding the use and duration of androgen deprivation therapy (ADT) were, as a general principle, based on the initial risk stratification group according to the NCCN Clinical Practice Guidelines [10]. Specifically, the CTV included only the prostate for low‐risk patients, the prostate plus approximately 1 cm of the proximal seminal vesicles (SV) for intermediate‐risk patients and the prostate plus the entire SV for high‐risk patients.

Following salvage radiotherapy, patients were monitored for PSA levels and clinical outcomes, including biochemical progression‐free survival, local control, overall survival and cancer‐specific survival. During the initial 1–2 years after radiation therapy, patients were typically monitored with PSA measurements, physical examinations and symptom assessments every 3–6 months. From 2 to 5 years, PSA measurements and clinical evaluations were generally conducted every 6–12 months, with further imaging recommended if there was a suspicion of recurrence. Beyond 5 years, annual PSA measurements and clinical assessments were performed. Toxicity was assessed using the Common Terminology Criteria for Adverse Events (CTCAE) version 5.0, with a focus on urinary and bowel function.

## 3 Results

A total of eight patients who underwent salvage radiotherapy following HIFU for localised prostate cancer were included in this analysis. Patient characteristics and clinical outcomes are presented in Table 1. All patients were male with an Eastern Cooperative Oncology Group Performance Status score of 0. The median age at the time of salvage radiotherapy was 66 years (range: 56–73 years), and the median time from HIFU to biochemical recurrence was 45.6 months (range: 18.6–110 months). Magnetic resonance imaging data were available for 7 out of 8 patients and were used to determine the clinical stage. TNM stage classifications were predominantly T1c and T2a, with one patient at T3 and one patient lacking TNM staging information. Grade Groups ranged from 1 to 5. Two patients with Grade Group 3 prostate cancer received short‐term ADT for 6 months before and during radiotherapy. Two patients with Grade Group 4 or higher were administered long‐term ADT, consisting of short‐term ADT followed by an additional 1.5 years of ADT after radiotherapy. The modality of EBRT mainly involved a conventional dose of 76 Gy delivered in 38 fractions. However, two patients received SABR with doses ranging from 36.25 to 40 Gy in five fractions. One SABR patient used a rectal spacer placed between the prostate and rectum to reduce the risk of radiation‐induced rectal injury. The other patient was not a candidate for this intervention due to being in clinical T3 stage, which indicates that the tumour has grown outside the prostate.

**TABLE 1:** Patient characteristics and outcomes following salvage radiotherapy after high‐intensity focused ultrasound for recurrent localised prostate cancer.

Age	Risk group before HIFU and PSA	HIFU‐to‐salvage RT (months)	PSA after HIFU and before salvage RT (ng/mL)	T staging	Grade group	ADT	Dose and fractionation	Observation periods (months)	Final status
66	High (PSA: 11.0)	18.6	1.8	N.A.	5	2y	76 Gy/38Fr	137.0	Survival (N.E.R.)
71	Intermediate (PSA: 11.4)	45.3	11.7	T1c	1	No	76 Gy/38Fr	125.0	Survival (N.E.R.)
70	Intermediate (PSA: 9.3)	46.0	5.5	T1c	2	No	76 Gy/38Fr	113.0	Survival (N.E.R.)
63	Intermediate (PSA: 17.5)	62.1	11.2	T1c	3	0.5y	76 Gy/38Fr	80.8	Survival (N.E.R.)
66	Intermediate (PSA: 8.7)	44.2	2.8	T2a	2	No	76 Gy/38Fr	12.3	Survival (N.E.R.)
64	Intermediate (PSA: 4.1)	64.6	4.2	T1c	2	No	76 Gy/38Fr	60.3	Survival (N.E.R.)
73	N.A.	110.2	7.0	T2a	3	0.5y	36.25 Gy/5Fr	64.0	Survival (N.E.R.)
56	N.A.	22.1	12.8	T3	4	2y	40 Gy/5Fr	58.6	Survival (N.E.R.)

Abbreviations: ADT, androgen deprivation therapy; HIFU, high‐intensity focused ultrasound; N.A., not assessed; N.E.R., no evidence of recurrence; PSA, prostate‐specific antigen; RT, radiotherapy.

All 8 patients showed a significant decline in PSA levels and remained free from biochemical failure during a median follow‐up of 72.4 months (range: 12.3–137 months). All patients were alive at the time of the last follow‐up, and no cancer‐specific deaths were recorded. The 5‐year biochemical failure‐free survival and overall survival rates were both 100%.

Four patients experienced acute Grade 1 urinary toxicity, primarily presenting as increased frequency and urgency, which resolved with symptomatic management. Two patients reported Grade 2 urinary toxicity, and no late Grade 3 or higher urinary toxicity was observed. Acute Grade 1 bowel toxicity, including diarrhoea and rectal discomfort, was reported in five patients and managed with symptomatic treatment. Three patients reported Grade 2 bowel toxicity, and no late Grade 3 or higher toxicity was observed. No Grade 3 or higher urinary or bowel toxicity was observed as a late adverse event.

## 4 Discussion

The results of this study demonstrate that definitive EBRT is a viable salvage treatment option for localised prostate cancer patients who experience recurrence after HIFU therapy. In terms of toxicity, the adverse effects observed in patients undergoing salvage EBRT were all within Grade 1 and 2 genitourinary and gastrointestinal toxicities regarding acute and late phase events. The findings in the present study align with previous studies, suggesting that EBRT offers a promising alternative when primary treatment with HIFU fails, providing both biochemical control and favourable long‐term outcomes (Table 2). Lalla et al. conducted a retrospective analysis on 12 patients treated with EBRT after HIFU failure. At a median follow‐up of 46 months, the 5‐year biochemical failure‐free survival and metastasis‐free survival were both 83.3%, with overall survival (OS) at 100% [11]. Mesci et al. reviewed outcomes for 14 men who received salvage radiotherapy after HIFU treatment failure for prostate cancer, using data from 2009 to 2017. The analysis found that while salvage radiotherapy was well tolerated with no severe toxicity, 50% of patients experienced secondary biochemical failure at a median follow‐up of 54 months [12]. Cafuta et al. evaluated 13 patients, with a median age of 80 years, who underwent conventional fractionated salvage radiotherapy with a median dose of 73.6 Gy. After a median follow‐up of 76 months, biochemical recurrence occurred in 23.1% of patients at a median of 36.4 months with no severe gastrointestinal or genitourinary toxicity [13].

**TABLE 2:** Summary of relevant published series on salvage radiotherapy following HIFU failure in localised prostate cancer.

Author	Published year	Number of patients	Radiation therapy type	Median follow‐up (months)	Clinical outcome	ADT administration	Acute AE	Late AE
Lalla et al. [11]	2022	12 patients	Conventional fractionation (range: 76–78 Gy, *n* = 11) or MHRT (66 Gy in 22 fractions, *n* = 1)	46	83.3% (5 years BRFS)	9 patients (75%)	GU: Grade 1 (5 patients, 41.7%) GI: Grade 1 (2 patients, 16.7%)	GU/GI: no grade ≥ 2 events reported
Mesci et al. [12]	2022	14 patients	Conventional fractionation (median 71 Gy)	54	36% (5 years BRFS)	7 patients (50%)	GU: Grade 2 (3 patients), Grade 1 (3 patients) GI: Grade 2 (1 patient), Grade 1 (2 patients).	GU: Grade 2 (1 patient), Grade 1 (1 patient) GI: Grade 2 (1 patient), Grade 1 (2 patients)
Cafuta et al. [13]	2022	13 patients	Conventional fractionation (median 73.6 Gy)	76	Three patients experienced biochemical recurrence	6 patients (46%)	GU (cystitis): Grade 2 (2 patients) GI (diarrhoea): Grade 2 (1 patient)	GU (cystitis): Grade 2 (3 patients) GI (diarrhoea): Grade 2 (1 patient)
Rigo et al. [14]	2020	24 patients	MHRT (range: 71.4–74.2 Gy in 28 fractions, *n* = 16) or SABR (range: 30–35 Gy in 5 fractions, *n* = 8)	28	Complete local control of disease was achieved in 23/24 patients (96%)	5 patients (21%)	GI: Grade 2 (3 patients, actinic proctitis) GU: Grade 1 (12 patients)	—
Alongi et al. [15]	2014	15 patients	MHRT (median 71.4 Gy in 28 fractions)	12	Three patients experienced biochemical recurrence	7 patients (47%)	GU: Grade 1 (7 patients), Grade 2 (4 patients) GI (bowel): Grade 1 (4 patients), Grade 2 (1 patients)	No grade ≥ 3 late toxicities reported
Ripert et al. [16]	2012	6 patients	Conventional fractionation (74 Gy in 37 fractions)	36.5	83.3% (5 years BRFS)	0 patients (0%)	—	GU: Grade 1 (2 patients) GI: Grade 1 (1 patients)
Riviere et al. [17]	2010	100 patients	Conventional fractionation (median 72 Gy)	33	72.5% (5 years BRFS) (for the 83 patients treated with exclusive radiation therapy)	17 patients (17%)	GU: Grade 3 (3 patients), Grade 2 (29 patients), Grade 1 (22 patients) GI: Grade 2 (15 patient), Grade 1 (24 patients)	GU: Grade 2 (23 patients), Grade 1 (24 patients) GI: Grade 2 (10 patient), Grade 1 (2 patients)

Abbreviations: ADT, androgen deprivation therapy; AE, adverse event; BRFS, biochemical recurrence‐free survival; GI, gastrointestinal; GU, genitourinary; MHRT, moderately hypofractionated radiotherapy; SABR, stereotactic ablative radiotherapy.

One of the key strengths of this study is the high rate of disease control, with 100% of patients showing no evidence of biochemical recurrence on serum PSA evaluation. Additionally, our study included two cases of SABR, which did not result in any severe adverse events during the observation period. SABR is an emerging and promising modality that has proven effective and feasible for localised prostate cancer [18, 19].

Salvage radical prostatectomy is also commonly used for patients with recurrent prostate cancer following HIFU therapy as whole prostate gland therapy. Blank et al. conducted a meta‐analysis of twelve studies involving 482 patients to examine the outcomes of salvage radical prostatectomy in patients with prostate cancer recurrence after focal therapy failure. The median time of a follow‐up period was 27 months; biochemical recurrence was seen in 23% [20]. Spitznagel et al. [21] compared salvage robotic‐assisted laparoscopic radical prostatectomy after HIFU treatment to primary robotic‐assisted laparoscopic radical prostatectomy. They reported that the early oncological outcome of salvage robotic‐assisted laparoscopic radical prostatectomy is satisfactory; however, salvage cases have a higher rate of complication compared to the primary surgery group. Salvage brachytherapy is a recognised treatment option for recurrent prostate cancer after definitive EBRT [22]. However, its role in the setting of HIFU failure remains unclear. To the best of our knowledge, no studies have evaluated the safety or efficacy of salvage brachytherapy specifically after HIFU treatment.

This study is limited by its small sample size and retrospective design, which may impact the generalisability of the findings. Additionally, there was heterogeneity in the EBRT modalities used, including conventional methods and SABR. While the follow‐up period was sufficient to assess clinical outcomes within 5 years, longer follow‐up is necessary to evaluate long‐term outcomes and late disease recurrence. Despite these limitations, this analysis offers valuable insights into the use of EBRT after HIFU and supports its role as a salvage option for patients with localised prostate cancer recurrence.

In conclusion, salvage radiotherapy for recurrence after HIFU treatment for localised prostate cancer provides effective disease control with a manageable toxicity profile. The findings also suggest that administering ADT based on risk classification at the time of recurrence may be beneficial. These results indicate that salvage radiotherapy is a viable option for patients with recurrent disease post‐HIFU, with most patients achieving durable disease control and experiencing minimal severe side effects.

Future research with larger cohorts and longer follow‐up is necessary to better define the optimal treatment strategy for patients with recurrence after HIFU. Prospective trials comparing different salvage modalities would also help provide clearer guidance on the best management approach for these patients.

## Conflicts of Interest

The authors declare no conflicts of interest.

## Data Availability

The data that support the findings of this study are available on request from the corresponding author. The data are not publicly available due to privacy or ethical restrictions.

## References

[1] N. D. James , I. Tannock , J. N'Dow , et al., “The Lancet Commission on Prostate Cancer: Planning for the Surge in Cases,” Lancet 403 (2024): 1683–1722, 10.1016/s0140-6736(24)00651-2. 38583453 PMC7617369

[2] T. J. Wilt , K. E. Ullman , E. J. Linskens , et al., “Therapies for Clinically Localized Prostate Cancer: A Comparative Effectiveness Review,” Journal of Urology 205 (2021): 967–976, 10.1097/ju.0000000000001578. 33350857

[3] J. S. Hopstaken , J. G. R. Bomers , M. J. P. Sedelaar , M. Valerio , J. J. Fütterer , and M. M. Rovers , “An Updated Systematic Review on Focal Therapy in Localized Prostate Cancer: What Has Changed Over the Past 5 Years?,” European Urology 81 (2022): 5–33, 10.1016/j.eururo.2021.08.005. 34489140

[4] J. Javier‐DesLoges , M. A. Dall'Era , W. Brisbane , et al., “The State of Focal Therapy in the Treatment of Prostate Cancer: The University of California Collaborative (UC‐Squared) Consensus Statement,” Prostate Cancer and Prostatic Diseases (2024): 579–581, 10.1038/s41391-023-00702-1. 37553435

[5] H. Ayerra Perez , J. F. Barba Abad , and J. Extramiana Cameno , “An Update on Focal Therapy for Prostate Cancer,” Clinical Genitourinary Cancer 21 (2023): 712.e711–e718., 10.1016/j.clgc.2023.04.013. 37258359

[6] V. S. Bachu , J. Kedda , I. Suk , J. J. Green , and B. Tyler , “High‐Intensity Focused Ultrasound: A Review of Mechanisms and Clinical Applications,” Annals of Biomedical Engineering 49 (2021): 1975–1991, 10.1007/s10439-021-02833-9. 34374945 PMC8608284

[7] A. Bakavicius , G. Marra , P. Macek , et al., “Available Evidence on HIFU for Focal Treatment of Prostate Cancer: A Systematic Review,” International Brazilian Journal of Urology 48 (2022): 263–274, 10.1590/s1677-5538.Ibju.2021.0091. 34003610 PMC8932027

[8] T. M. Morgan , S. A. Boorjian , M. K. Buyyounouski , et al., “Salvage Therapy for Prostate Cancer: AUA/ASTRO/SUO Guideline Part III: Salvage Therapy After Radiotherapy or Focal Therapy, Pelvic Nodal Recurrence and Oligometastasis, and Future Directions,” Journal of Urology 211 (2024): 526–532, 10.1097/ju.0000000000003890. 38421252

[9] M. C. Abramowitz , T. Li , M. K. Buyyounouski , et al., “The Phoenix Definition of Biochemical Failure Predicts for Overall Survival in Patients With Prostate Cancer,” Cancer 112 (2008): 55–60, 10.1002/cncr.23139. 17968996

[10] E. M. Schaeffer , S. Srinivas , N. Adra , et al., “Prostate Cancer, Version 4.2023, NCCN Clinical Practice Guidelines in Oncology,” Journal of the National Comprehensive Cancer Network 21 (2023): 1067–1096, 10.6004/jnccn.2023.0050. 37856213

[11] V. Di Lalla , S. Elakshar , M. Anidjar , et al., “Salvage External Beam Radiotherapy After HIFU Failure in Localized Prostate Cancer: A Single Institution Experience,” Frontiers in Oncology 12 (2022): 1028858, 10.3389/fonc.2022.1028858. 36408146 PMC9669274

[12] A. Mesci , M. Gouran‐Savadkoohi , D. Ribeiro , et al., “Salvage Radiotherapy Following HiFU: An Institutional Series and Literature Review,” Journal of Medical Imaging and Radiation Oncology 66 (2022): 847–852, 10.1111/1754-9485.13384. 35170226

[13] B. Cafuta , F. A. Distler , A. Wagner , S. Pahernik , C. Albrecht , and G. Hatiboglu , “Salvage Radiotherapy for Recurrent Prostate Cancer After High‐Intensity Focused Ultrasound Therapy: Quality of Life and Functional Outcome,” Urologia Internationalis 106 (2022): 940–945, 10.1159/000521660. 35104820

[14] M. Rigo , R. Mazzola , G. Napoli , et al., “Post‐HIFU Locally Relapsed Prostate Cancer: High‐Dose Salvage Radiotherapy Guided by Molecular Imaging,” La Radiologia Medica 125 (2020): 491–499, 10.1007/s11547-020-01148-4. 32077006

[15] F. Alongi , R. L. Liardo , C. Iftode , et al., “11C Choline PET Guided Salvage Radiotherapy With Volumetric Modulation Arc Therapy and Hypofractionation for Recurrent Prostate Cancer After HIFU Failure: Preliminary Results of Tolerability and Acute Toxicity,” Technology in Cancer Research & Treatment 13 (2014): 395–401, 10.7785/tcrtexpress.2013.600268. 24000995 PMC4527415

[16] T. Ripert , Y. Bayoud , R. Messaoudi , et al., “Salvage Radiotherapy After High‐Intensity Focused Ultrasound Treatment for Localized Prostate Cancer: Feasibility, Tolerance and Efficacy,” Canadian Urological Association Journal 6 (2012): E179–E183, 10.5489/cuaj.10137. 21539766 PMC3478387

[17] J. Riviere , J. C. Bernhard , G. Robert , et al., “Salvage Radiotherapy After High‐Intensity Focussed Ultrasound for Recurrent Localised Prostate Cancer,” European Urology 58 (2010): 567–573, 10.1016/j.eururo.2010.06.003. 20598436

[18] T. A. C. Kennedy , W. L. Ong , H. Quon , et al., “Stereotactic Radiotherapy for Localized Prostate Cancer: 10‐Year Outcomes From Three Prospective Trials,” International Journal of Radiation Oncology, Biology, Physics 121 (2025): 325–330, 10.1016/j.ijrobp.2024.09.009. 39293531

[19] A. Katano , M. Minamitani , S. Ohira , and H. Yamashita , “Recent Advances and Challenges in Stereotactic Body Radiotherapy,” Technology in Cancer Research & Treatment 23 (2024): 15330338241229363, 10.1177/15330338241229363. 38321892 PMC10851756

[20] F. Blank , M. Meyer , H. Wang , et al., “Salvage Radical Prostatectomy After Primary Focal Ablative Therapy: A Systematic Review and Meta‐Analysis,” Cancers (Basel) 15 (2023): 15, 10.3390/cancers15102727. PMC10216462 37345064

[21] T. Spitznagel , J. V. Hardenberg , F. A. Schmid , et al., “Salvage Robotic‐Assisted Laparoscopic Radical Prostatectomy Following Focal High‐Intensity Focused Ultrasound for ISUP 2/3 Cancer,” Urology 156 (2021): 147–153, 10.1016/j.urology.2021.04.059. 34186136

[22] M. T. Corkum , M. K. Buyyounouski , A. J. Chang , et al., “Salvage Prostate Brachytherapy in Radiorecurrent Prostate Cancer: An International Delphi Consensus Study,” Radiotherapy and Oncology 184 (2023): 109672, 10.1016/j.radonc.2023.109672. 37059334

